# THE ROLE OF PERSONALITY IN RETIREMENT ADJUSTMENT: LONGITUDINAL EFFECTS ON LIFE SATISFACTION

**DOI:** 10.1093/geroni/igz038.080

**Published:** 2019-11-08

**Authors:** Isabelle Hansson, Georg Henning, Sandra Buratti, Magnus Lindwall, Marie Kivi, Boo Johansson, Anne Ingeborg Berg

**Affiliations:** 1 Department of Psychology and Centre for Ageing and Health (AgeCap), University of Gothenburg, Gothenburg, Sweden; 2 Department Of Psychology And Centre For Ageing And Health (AgeCap), University Of Gothenburg, Gothenburg, Vastra Gotaland, Sweden

## Abstract

Research on the retirement transition suggests that personality can influence the adjustment process, but the mechanisms involved remain still largely unknown. In the present study we investigate direct and indirect associations between the Big Five personality traits and life satisfaction over the retirement transition. Indirect effects were evaluated through the role of personality for self-esteem, autonomy, social support, perceived physical and cognitive health, and financial satisfaction. The sample included 796 older adults and four annual measurement waves in the Swedish longitudinal HEARTS study. Results from multivariate latent growth curve models showed multiple indirect effects of personality. Extraversion was positively related to life satisfaction through increased self-esteem, autonomy, and social support. Neuroticism was negatively associated with life satisfaction through decreased self-esteem, autonomy, social support, and perceived cognitive ability. Our findings suggest that retirees with higher neuroticism are more likely to experience adjustment problems resulting from negative changes in key resources.

